# Successful Surgical Resection for Protein-Losing Enteropathy Caused by Ischemic Ileitis

**DOI:** 10.70352/scrj.cr.26-0170

**Published:** 2026-06-17

**Authors:** Kazuki Ueda, Kei Harada, Ippei Yamana, Takahisa Fujikawa

**Affiliations:** Department of Surgery, Kokura Memorial Hospital, 3-2-1 Asano, Kokurakita-ku, Kitakyushu, Fukuoka 802-8555, Japan

**Keywords:** protein-losing enteropathy, ischemic ileitis, ileocecal resection

## Abstract

**INTRODUCTION:**

Protein-losing enteropathy (PLE) is a condition characterized by the excessive loss of serum proteins through the gastrointestinal mucosa, which causes hypoalbuminemia and edema. Common causes include inflammatory diseases, neoplastic diseases, lymphatic diseases, and cardiac diseases. However, cases of PLE caused by small bowel ischemia and cured by surgical resection are rare. Herein, we report a case of PLE caused by ischemic ileitis that was successfully treated by surgical resection.

**CASE PRESENTATION:**

A 76-year-old woman presented with diarrhea and edema. She had cardiovascular disease and had undergone an ileocecal resection for cecal cancer 15 years ago. Laboratory tests revealed severe hypoalbuminemia without hepatic or renal dysfunction. A contrast-enhanced CT scan revealed edema and panniculitis in the terminal ileum. Technetium-99m-labeled human serum albumin scintigraphy revealed focal protein leakage in the terminal ileum. We diagnosed the patient with PLE caused by ischemic ileitis, and conservative treatment was ineffective, so we performed a resection of the terminal ileum and created an ileostomy. Histopathological examination revealed mucosal erosions, ulcers, and submucosal fibrosis consistent with ischemic ileitis. No malignancy was found. After surgery, serum albumin levels normalized over time, and the patient was discharged after ilieostomy closure.

**CONCLUSIONS:**

Ischemic ileitis may lead to PLE, particularly in patients with underlying vascular risk factors. Surgical resection may be effective when the causative lesion is localized and conservative treatment is unsuccessful.

## Abbreviation

PLE
: protein-losing enteropathy

## INTRODUCTION

PLE is caused by excessive leakage of plasma proteins from the gastrointestinal tract, resulting in hypoalbuminemia, edema, diarrhea, and malnutrition. The causes include inflammatory bowel disease, intestinal lymphangiectasia, malignancy, and congestive heart failure.^1,2)^ In many cases, conservative treatment or medical treatment for the underlying disease is chosen, but if the cause is a localized organic lesion, surgical resection may be effective.

Ischemic enteritis is a disease in which ischemic lesions occur in the small intestine due to intestinal microcirculatory disorders. Because the small intestine has abundant collateral vessels, ischemic lesions are unlikely to occur, and cases of ischemic enteritis complicated by PLE are rare.^3,4)^ Furthermore, cases of PLE caused by chronic small bowel ischemia and successfully treated by surgical resection are rare.

Herein, we report a case of PLE caused by ischemic ileitis that was successfully treated by surgical resection.

## CASE PRESENTATION

A 76-year-old woman visited our hospital complaining of several months of progressive diarrhea and bilateral lower limb edema. She had undergone ileocecal resection for cecal cancer 15 years ago. She had a history of cardiovascular disease, including aortic dissection and aortic valve stenosis, and was undergoing regular outpatient follow-up.

Laboratory tests revealed marked hypoalbuminemia. Liver enzyme levels and renal function parameters were within normal limits, and urinalysis revealed no proteinuria. There was no clinical or biochemical evidence of liver dysfunction, nephrotic syndrome, or systemic inflammatory disease (**Table 1**). A contrast-enhanced CT scan revealed focal wall thickening, edema, and increased fatty tissue density in the terminal ileum (**Fig. 1**). There was no tumor recurrence, lymphadenopathy, or major mesenteric vascular occlusion. Endoscopic examination revealed that only a small amount of mucosa remained around the anastomosis in the ileum on the oral side of the anastomosis after ileocecal resection, and the villus structure was barely visible. Furthermore, circumferential mucosal delamination and ulcer formation were observed in the ileum proximal to the anastomosis site (**Fig. 2**). Technetium-99m (^99m^Tc)-labeled human serum albumin scintigraphy revealed what appeared to be extravascular accumulation in the midline and right lower quadrant of the abdomen, and the accumulation in these areas was found to spread over time along the course of the gastrointestinal tract (**Fig. 3**).

**Table 1:** Patient blood biochemistry profile

Parameter	Result	Reference range
CRP	2.1 mg/L	0.0–0.14 mg/L
WBC	6.1 × 10^3^/μL	3.3–8.6 × 10^3^/μL
BUN	13.0 mg/dL	8.0–20.0 × 10^3^/μL
Creatinine	0.54 mg/dL	0.46–0.79 mg/dL
Hemoglobin	9.9 g/dL	11.6–14.8 g/dL
Platelet	21.6 × 10^4^/μL	15.8–34.8 × 10^4^/μL
AST	19 U/L	13–30 U/L
ALT	17 U/L	7–23 U/L
ALP	109 U/L	106–322 U/L
TP	4.0 g/dL	6.6–8.1 g/dL
Albumin	1.1 g/dL	4.1–5.1 g/dL
PT	74%	70%–130%
APTT	34.5 s	24–39 s
D-dimer	17.1 ng/mL	<1.0 ng/mL
sIL-2R	814 U/L	122–496 U/mL
ssDNA-G	9.4 AU/mL	<25.0 AU/mL
IgG	1313 mg/dL	861–1747 mg/dL
PR3-ANCA	<1.0 IU/mL	—
MPO-ANCA	<1.0 IU/mL	—
Antinuclear antibody	Negative	—
CMV pp65 Ag	Negative	—
Tb IFN-γ	Negative	—

ALP, alkaline phosphatase; ALT, alanine aminotransferase; APTT, activated partial thromboplastin time; AST, aspartate aminotransferase; BUN, blood urea nitrogen; CMV pp65 Ag, cytomegalovirus pp65 antigenemia; CRP, C-reactive protein; IgG, immunoglobulin G; MPO-ANCA, myeloperoxidase anti-neutrophil cytoplasmic antibody; PR3-ANCA, proteinase 3-antineutrophil cytoplasmic antibody; PT, prothrombin time; sIL-2R, soluble interleukin-2 receptor; ssDNA-G, anti-single stranded DNA IgG; Tb IFN-γ, interferon-gamma release assay for tuberculosis; TP, total protein; WBC, white blood cell count

**Fig. 1:**
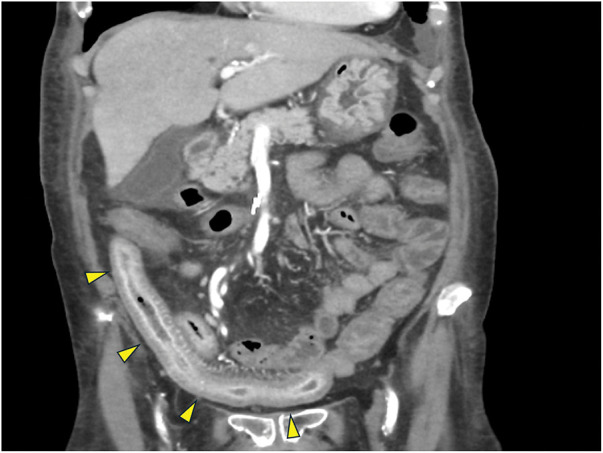
Contrast-enhanced CT shows focal wall thickening and edema of the terminal ileum (arrowheads).

**Fig. 2:**
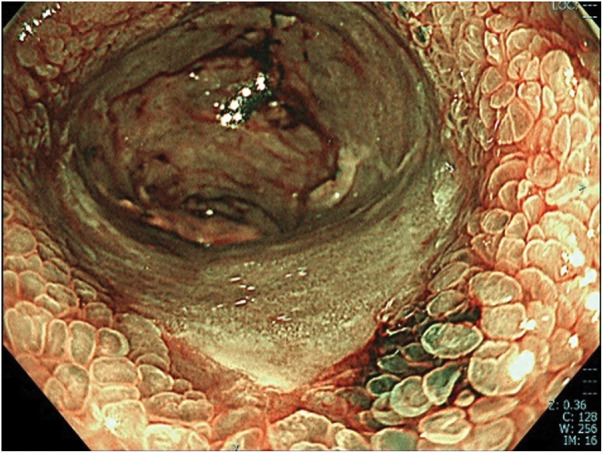
Endoscopic examination revealed that only a small amount of mucosa remained in the ileum on the oral side of the anastomosis after ileocecal resection, and the mucosal epithelium had completely fallen off, making it impossible to confirm the villus structure. Furthermore, circumferential mucosal delamination and ulcer formation were observed in the ileum proximal to the anastomosis site.

**Fig. 3:**
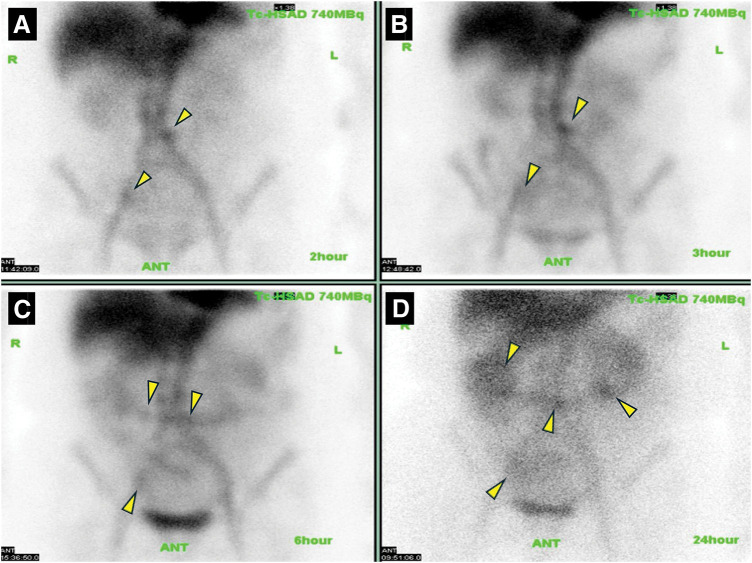
Technetium-99m-labeled human serum albumin scintigraphy revealed that extravascular accumulation (yellow arrowheads) was observed in the midline and right lower quadrant of the abdomen starting 2 h after administration, and accumulation in these areas was observed to spread over time along the course of the gastrointestinal tract (**A** → **B** → **C** → **D**).

Based on these findings, a diagnosis of PLE due to ischemic ileitis was made. Conservative treatment, including fasting, total parenteral nutrition, and albumin supplementation, did not adequately improve diarrhea and hypoalbuminemia until the 16th day of hospitalization. Because the protein-leaking lesion was localized and the symptoms persisted, surgical resection using intraoperative endoscopy was planned and performed on the 51st day of hospitalization. In addition, in preparation for the surgery, the patient received daily albumin administration starting before the surgery. Laparotomy revealed focal wall thickening in the terminal ileum and an irregular, nodular ileal serosal surface, suggestive of chronic ischemic changes (**Fig. 4**). No gross tumor was observed. Using intraoperative endoscopy, observation was performed from the serosal and mucosal surfaces, and resection of the terminal ileum, which was considered to be the culprit lesion, was performed. Given the patient's hypoalbuminemia and the associated risk of anastomotic complications, a staged surgical approach was adopted. A temporary stoma was created during the initial resection, and the stoma was scheduled to be closed after nutritional recovery.

**Fig. 4:**
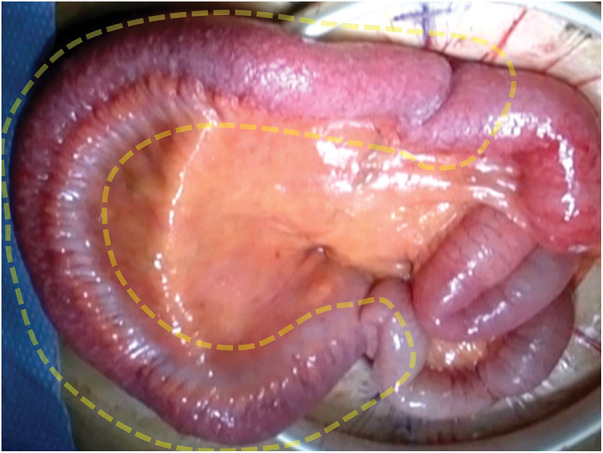
Laparotomy revealed focal wall thickening in the terminal ileum and an irregular, nodular ileal serosal surface.

The resected specimen showed localized wall thickening and ulceration in the terminal ileum, starting approximately 2.5 cm from the anastomosis site of the previous surgery (**Fig. 5**). Histopathological examination revealed that epithelial cells had fallen off and villi had atrophied in the ulcerated areas and rough mucosal regions, and granulation tissue and fibrosis were observed in the lamina propria and submucosa (**Fig. 6**). No findings suggestive of malignancy were found.

**Fig. 5:**
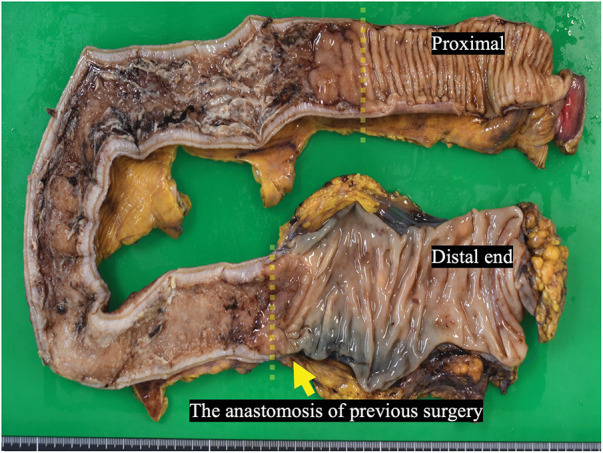
Macroscopic examination of the tissue revealed widespread ulceration on the ileal wall, bordered by a yellow dotted line.

**Fig. 6:**
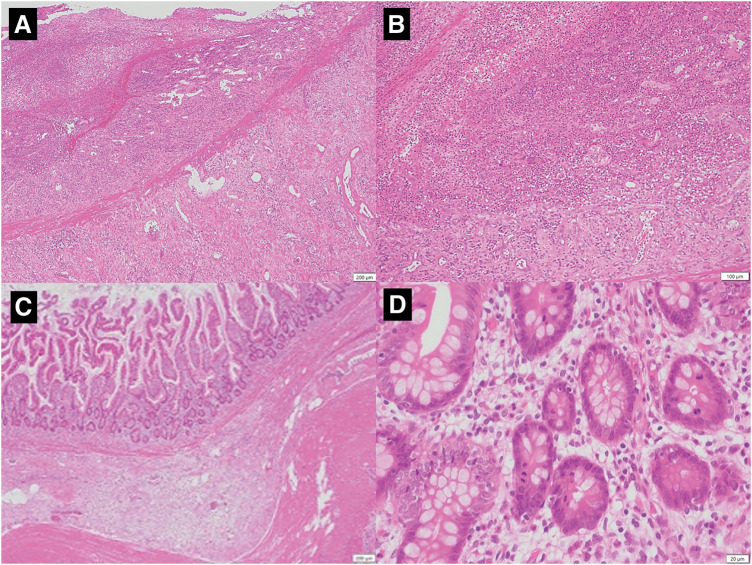
In mucosal areas with ulceration and surface roughening, epithelial delamination, villous atrophy, granulation tissue formation, and fibrosis were observed in the lamina propria and submucosa, accompanied by infiltration of neutrophils, lymphocytes, and plasma cells (**A**, **B**). Inflammation and fibrosis extended to the subserosa (**C**). Foreign-body multinucleated giant cells were observed within the mucosa (**D**).

After surgery, diarrhea and peripheral edema gradually improved, and serum albumin levels rapidly increased (**Fig. 7**). After the patient's nutritional status was restored, the ileostomy was closed.

**Fig. 7:**
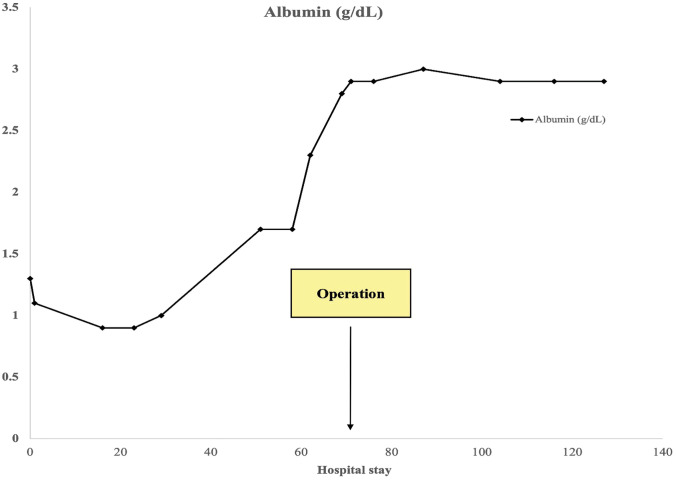
Changes in Alb levels during the clinical course. After surgery, Alb levels rapidly increased. Before surgery, Alb transfusions were administered as needed. Alb, albumin

## DISCUSSION

This case is significant because it demonstrates that ischemic ileitis can cause PLE and that surgical resection may lead to a cure when the causative lesion is localized and conservative treatment is ineffective.

Although important collateral circulation exists in the terminal ileum, distal ileal ischemic injury has been reported in cases of mesenteric dialysis circulation impairment, and it is a rare but possible occurrence.^5)^ Approximately half of the cases have been shown to have underlying arteriosclerotic diseases such as hypertension, ischemic heart disease, arrhythmia, cerebrovascular disease, and diabetes.^3,6,7)^ These facts suggest that systemic vascular factors and local anatomical factors may be involved in a complex manner. Although this case did not show any obvious occlusion of the major mesenteric artery, the patient had a history of cardiovascular disease. Even without obvious vascular occlusion, hypoperfusion caused by factors such as arteriosclerosis, microcirculatory disorders, and cardiac dysfunction can lead to chronic mesenteric ischemia.^8)^ The patient had undergone ileocecal resection 15 years prior, but a direct causal relationship between the previous surgery and the development of ischemic ileitis could not be established. Therefore, the previous surgery should be considered as part of the patient’s clinical background, rather than as a proven etiology. In this case, underlying cardiovascular disease and chronic hypoperfusion may have contributed to the ischemic mucosal injury.

The main pathogenesis of PLE is the disruption of mucosal barrier function and increased permeability due to ischemia.^2)^ The pathological findings in this case supported these chronic ischemic changes. While these pathological findings were not specific to PLE, they suggest severe mucosal damage, ulceration, inflammation, and fibrosis, which may explain protein leakage from the affected intestinal tract. In the diagnosis, ^99m^Tc-labeled human serum albumin scintigraphy was able to localize the site of protein leakage, which was useful in determining whether surgical resection was appropriate. PLE is primarily treated with medical therapy and treatment of the underlying disease.^1,2)^ In some cases, as in this case, surgical resection is the definitive treatment.^9,10)^ Surgical intervention should be considered when ischemic ileitis causes PLE despite conservative treatment, and the causative lesion is localized. In severe circumferential ischemic lesions, progressive ulcer formation can lead not only to protein leakage but also to impaired bowel motility, bile acid malabsorption, refractory diarrhea, and ultimately stricture formation. These characteristics suggest that long-term conservative treatment may even be insufficient in some patients. In this case, the combination of severe hypoalbuminemia, persistent diarrhea, circumferential ulceration, and localized protein leakage led to the conclusion that surgical resection was the appropriate curative treatment. Furthermore, it is worth noting that a 2-stage operation was chosen due to the risk of anastomotic rupture associated with the primary anastomosis in severe hypoalbuminemia. Hypoalbuminemia is considered an independent risk factor for delayed wound healing and anastomotic leakage.^11)^ In the initial operation, we thoroughly resected the culprit lesion and created a temporary stoma, minimizing the risk of complications. Afterward, we safely closed the stoma after waiting for the patient's nutritional status to improve. This strategy suggests the possibility of a highly reproducible approach for surgical intervention for PLE caused by ischemic ileitis.

This case demonstrates that ischemic ileitis can cause PLE in patients with underlying cardiovascular and arteriosclerotic disease. When unexplained hypoalbuminemia is present along with persistent diarrhea and localized small bowel abnormalities, PLE due to ischemic ileitis should be considered, and identifying the location of the protein-losing lesion may help determine whether surgical treatment is appropriate.

## CONCLUSIONS

We have experienced a case in which surgical resection was successful for PLE caused by ischemic ileitis. In patients with underlying cardiovascular disease who present with persistent gastrointestinal symptoms and unexplained hypoalbuminemia, ischemic ileitis with PLE should be considered. In some cases, localization of the protein-losing lesion and surgical intervention may be effective.

## DECLARATIONS

### Availability of data and materials

The datasets supporting the conclusions of this article are included within the article.
